# Supplementary material for: Engineered Glucose Oxidase Capable of Quasi-Direct Electron Transfer after a Quick-and-Easy Modification with a Mediator
Source: Int J Mol Sci. 2020 Feb 8;21(3):1137. doi: 10.3390/ijms21031137 (PMC7036908; doi:10.3390/ijms21031137)
Supplement: Supplementary file 1 [file ijms-21-01137-s001.pdf]

Supplementary Materials

Engineered Glucose Oxidase Capable of Quasi-Direct Electron Transfer after Quick-and-Easy Modification with a Mediator

Nanami Suzuki [1,†], Jinhee Lee [2,†], Noya Loew [2], Yuka Takahashi-Inose [3], Junko Okuda-Shimazaki [2], Katsuhiro Kojima [3], Kazushige Mori [3], Wakako Tsugawa [1] and Koji Sode [2,*]

1 Department of Biotechnology and Life Science, Graduate School of Engineering, Tokyo University of Agriculture and Technology, 2-24-16 Naka-cho, Koganei, Tokyo, 184-8588, Japan; nsuzuki@protonmail.com (N.S.); tsugawa@cc.tuat.ac.jp (W.T.)

2 Joint Department of Biomedical Engineering, The University of North Carolina at Chapel Hill and North Carolina State University, Chapel Hill, NC 27599, USA; jh.lee@unc.edu (J.L.); noya-loew@rs.tus.ac.jp (N.L.); jokudas@email.unc.edu (J.O.-S.); ksode@email.unc.edu (K.S.)

3 Ultizyme International Ltd., 3-9-5. Taihei, Sumida, Tokyo, 130-0012, Japan; yukappe1122@gmail.com (Y.T.-I.); katsuhiro.kojima@gmail.com (K.K.); mori_ka1213@yahoo.co.jp (K.M.)

* Correspondence: ksode@email.unc.edu; Tel.: +1-919-966-3550

† These authors contributed equally to this work.

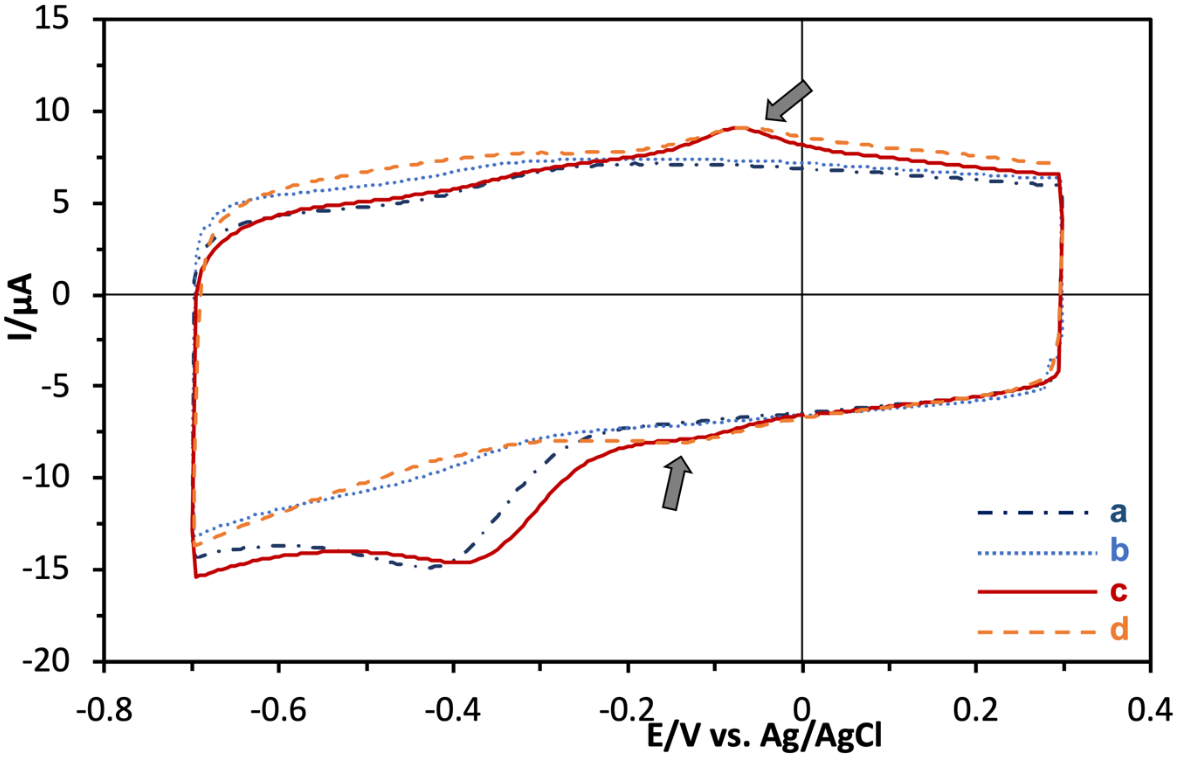

Figure S1. *Cyclic voltammograms of (a) unmodified AnGOx-WT in the absence of glucose; (b) unmodified AnGOx-WT in presence of 33 mM glucose; (c) PES-modified AnGOx-WT in absence of glucose; (d) PES-modified AnGOx-WT in presence of 33 mM glucose. Scan rate 50 mV/s. Arrows: peaks due to PES.*

Table S1. *Kinetic parameters of AnGOx-WT and AnGOx-I489K*

Enzyme	PES modification	Oxidase Activity			Dehydrogenase Activity		
		V_{max} (U/mg)	K_m (mM)	V_{max}/K_m (U/mg·mM)	V_{max} (U/mg)	K_m (mM)	V_{max}/K_m (U/mg·mM)
*An*GOx-WT	−	140	36	3.8	50	32	1.6
	+	170	40	4.3	53	26	2
*An*GOx-I489K	−	76	28	2.7	22	22	1
	+	130	42	3	49	23	2.2

Oxidase activity was determined by monitoring the increase of absorbance at 555 nm (formation of quinoeimine dye) of a mixture of GOx, glucose (var. concentrations), 1.5 mM 4-aminoantipyrine (FUJIFILM Wako Pure Chemical Corporation, Osaka, Japan), 1.5 mM *N*-ethyl-*N*-(2-hydroxy-3-sulfopropyl)-3-methylaniline (Dojindo Laboratories Co. Ltd., Kumamoto, Japan), and 2 U/mL horseradish peroxidase (HRP) (Amano enzyme Inc., Gifu, Japan) in 20 mM P.P.B.. Quinoeimine dye is formed by HRP in presence of hydrogen peroxide. The formation of 1 μmol/min hydrogen peroxide, corresponding to the oxidation of 1 μmol/min glucose, was defined as 1 U oxidase activity.

Dehydrogenase activity was determined by monitoring the decrease of absorbance at 600 nm (reduction of 2,6-dichlorophenolindophenol, DCIP (Kanto Chemical Co. Inc., Tokyo, Japan)) of a mixture of GOx, glucose (var. concentrations), 0.6 mM PMS, and 0.06 mM DCIP in 20 mM P.P.B. The reduction of 1 μmol/min DCIP, corresponding to the oxidation of 1 μmol/min glucose, was defined as 1 U dehydrogenase activity.
